# Long-Term pemafibrate treatment exhibits limited impact on body fat mass in patients with hypertriglyceridemia accompanying NAFLD

**DOI:** 10.3389/fendo.2024.1329294

**Published:** 2024-05-17

**Authors:** Takanobu Iwadare, Takefumi Kimura, Hideo Kunimoto, Taiki Okumura, Shun-Ichi Wakabayashi, Hiroyuki Kobayashi, Yuki Yamashita, Ayumi Sugiura, Naoki Tanaka, Takeji Umemura

**Affiliations:** ^1^ Department of Medicine, Division of Gastroenterology and Hepatology, Shinshu University School of Medicine, Matsumoto, Japan; ^2^ Department of Gastroenterology, Nagano Municipal Hospital, Nagano, Japan; ^3^ Consultation Center for Liver Diseases, Shinshu University Hospital, Matsumoto, Japan; ^4^ Department of Global Medical Research Promotion, Shinshu University Graduate School of Medicine, Matsumoto, Japan; ^5^ International Relations Office, Shinshu University School of Medicine, Matsumoto, Japan; ^6^ Research Center for Social Systems, Shinshu University, Matsumoto, Japan

**Keywords:** non-alcoholic fatty liver disease, hypertriglyceridemia, pemafibrate, selective PPARα modulator, body composition analysis, treatment response, metabolic dysfunction-associated steatotic liver disease

## Abstract

**Aim:**

Short-term use of pemafibrate (PEM), a selective modulator of peroxisome proliferator-activated receptor alpha, has been reported to improve abnormal liver function in patients with nonalcoholic fatty liver disease with hypertriglyceridemia (HTG-NAFLD). This study aimed to clarify the effects and predictive factors of long-term 72-week PEM administration on body composition, and laboratory tests in HTG-NAFLD patients.

**Methods:**

Fifty-three HTG-NAFLD patients receiving a 72-week PEM regimen were retrospectively enrolled. Routine blood and body composition results were analyzed immediately before and at the end of the study period.

**Results:**

PEM treatment significantly improved liver enzyme levels such as aspartate aminotransferase (AST), alanine aminotransferase (ALT), alkaline phosphatase, and gamma-glutamyl transferase, along with lipid profiles including triglyceride, total cholesterol, and low-density lipoprotein cholesterol. PEM did not have any detectable impact on body composition parameters. The factors of female, higher AST (≥ 46 U/L) and fat mass (≥ 31.9%), as well as lower soft lean mass (< 61.6%), skeletal muscle mass (< 36%), and skeletal muscle mass index (< 6.9 kg/m^2^) were significantly associated with the treatment response status of a > 30% decrease in ALT. All patients completed the treatment without any adverse effects.

**Conclusions:**

Long-term PEM treatment had a positive impact on liver enzymes and lipid profiles, but it did not result in significant changes in body composition among HTG-NAFLD patients. In predicting the response to PEM treatment, the evaluation of AST and body composition may be useful.

## Introduction

Non-alcoholic fatty liver disease (NAFLD) is a chronic liver condition characterized by the accumulation of triglyceride (TG) in over 5% of the liver (1). NAFLD has emerged as the most prevalent liver disease worldwide, leading to significant morbidity and mortality. In fact, its global prevalence is surpassing previous estimates and continues to rise at an alarming pace (2). The progression from simple steatosis to non-alcoholic steatohepatitis (NASH) can lead to the development of liver cirrhosis and potentially hepatocellular carcinoma (HCC) (3).

While no specific pharmacological treatments are currently approved for NAFLD, ongoing clinical trials are investigating various drug candidates targeting energy intake, energy disposal, lipotoxic liver injury, inflammation, and fibrosis (4). One such candidate is pemafibrate (PEM), a selective peroxisome proliferator-activated receptor-alpha (PPARα) modulator recently approved in Japan in 2018 for dyslipidemia. To evaluate the pharmacological and toxicological effects of PEM, comprehensive transcriptome analyses have been conducted on primary human hepatocytes and mouse liver tissue, which showed the induction of PPARα target genes involved in key hepatic processes, including TG hydrolysis, fatty acid uptake, fatty acid β-oxidation, and ketogenesis (5). In animal NASH models, PEM administration produced notable improvements in obesity, dyslipidemia, liver dysfunction, and NASH-associated pathological features (6). In another recent study, PEM treatment led to improvements in liver function tests, fibrotic biomarkers, and FibroScan-AST (FAST) score in NAFLD with hypertriglyceridemia (HTG-NAFLD) patients, suggesting a potential to prevent disease progression (7).

Several studies in mice have investigated the effects of PEM on adipose tissue but provided conflicting results. Araki et al. reported that PEM activated thermogenesis in mouse inguinal white adipose tissue (iWAT) and brown adipose tissue (BAT) by increasing plasma fibroblast growth factor 21 (FGF21) levels. The drug induced the expression of adipose triglyceride lipase (Atgl) and hormone-sensitive lipase (Hsl) in epididymal white adipose tissue (eWAT), leading to lipolysis activation and a presumed ability to decease fat mass (8). On the other hand, Zhang reported that the administration of a clinical volume of PEM to mice increased FGF21 levels but did not alter fatty acid uptake, fatty acid synthesis, TG synthesis, lipolysis (Atgl, Hsl), fatty acid beta-oxidation, or browning genes in eWAT or BAT (9). These incongruent results raised clinical questions about the potential of PEM to reduce body fat mass. Although short-term PEM treatment of HTG-NAFLD patients did not impact body fat mass or other related body composition parameters in our earlier study (10), long-term observations were considered necessary to confirm the body compositional changes from PEM.

In this investigation, we sought to clarify the effects of long-term PEM treatment on body fat mass and laboratory tests including liver function and lipid profile in HTG-NAFLD patients, as well as exploring predictive factors for PEM treatment responsiveness.

## Materials and methods

### Patients and clinical examinations

This was a retrospective analysis of prospectively registered patients. The present case-control study was approved by Nagano Municipal Hospital (ID number: 0038) and was performed following the Helsinki declaration of 1975 (1983 revision). We prospectively registered the 53 Japanese NAFLD patients who treated with PEM at Nagano Municipal Hospital (Nagano, Japan) between September 2019 and April 2023. Body composition and blood test data before, 24 weeks, and 72 weeks after PEM administration were used to retrospectively examine the therapeutic effect of long-term PEM administration in the HTG-NAFLD patients. Additionally, we defined responders as those showing an improvement of ALT >30% at 72 weeks according to previous studies (10–12), and conducted an analysis to identify predictive factors for responders using blood test and body composition data obtained before PEM administration.

The inclusion criteria for the HTG-NAFLD patients were as follows: (1) presence of hepatorenal contrast and increased hepatic echogenicity on abdominal ultrasonography; and (2) fasting serum levels of TG > 150 mg/dL (13). The main exclusion criteria included patients with (1) average alcohol consumption of <30 g/day in men and <20 g/day in women; (2) absence of other causes of liver dysfunction, such as viral hepatitis, drug-induced liver injury, autoimmune liver disease, Wilson’s disease, hereditary hemochromatosis, and citrin deficiency (14). Before PEM commencement, all patients had well-preserved liver function (i.e., not Child-Pugh class B or C) and no signs of HCC, gallstones, or renal impairment (i.e., serum creatine [Cre] concentration ≥ 2.5 mg/dL).

Patients were defined as hypertensive if their systolic/diastolic pressure was > 140/90 mmHg or if they were taking anti-hypertensive drugs (15). Type 2 diabetes is diagnosed with a fasting plasma glucose level ≥126 mg/dL on two occasions, HbA1c ≥6.5%, OGTT plasma glucose ≥200 mg/dL after 2 hours, or random plasma glucose ≥200 mg/dL with classic symptoms of hyperglycemia or if they were taking insulin or oral hypoglycemic agents (16). The diagnosis of liver cirrhosis (LC) was based on imaging findings and the formula for predicting LC proposed by Ikeda et al. (17). All laboratory data and body composition measurements were obtained in a fasting state. Fibrosis-4 (FIB-4) was calculated according to the following formula: FIB-4 = (age [years] × AST [U/L])/(platelet count [x10^9^/L] × alanine aminotransferase [ALT] [U/L]^1/2^) (18). The interval between patient visits for blood sampling was 4-12 weeks, at which time the patient was also interviewed about side effects.

### Body composition analysis

Body composition analysis was conducted on 53 patients at the initiation of PEM administration using an InBodyS10 multi-frequency impedance body composition analyzer (InBody Japan, Tokyo). The analysis included measurements of fat mass, soft lean mass, and skeletal muscle (SKM) mass. Subsequently, 40 patients were assessed at 3 time points: immediately before PEM administration (baseline) and at 24 and 72 weeks later. Fat mass (%), soft lean mass (%), and SKM mass (%) were calculated by dividing the amounts calculated by body composition analysis by body weight. Skeletal muscle mass index (SMI) was determined as appendicular SKM mass (kg)/height (m)^2^.

### Statistical analysis

Clinical data are expressed as the number (percentage) or median (interquartile range). Statistical analyses were performed using StatFlex Ver. 7.0 (Artech Co., Ltd., Osaka, Japan). Wilcoxon matched-pairs signed-rank testing was employed for evaluating parameters before and after PEM treatment. The Friedman test was adopted for evaluating parameters before, at 24 weeks, and at 72 weeks of PEM treatment. The Mann–Whitney test and chi-square test were employed to compare responders and non-responders to PEM treatment. The diagnostic accuracy for identifying predictive factors of treatment responsiveness was assessed using the area under the receiver operating characteristic (ROC) curve (AUROC). AST, ALT, SKM mass (%), and SMI were employed as parameters in the ROC analysis of this study. The Youden index identified cut-off values, with the nearest clinically applicable value to the cut-off considered the optimal threshold for clinical convenience. All statistical tests were evaluated at the 0.05 level of significance.

## Results

### Clinical characteristics of HTG-NAFLD patients treated with PEM

53 patients completed the full 72-week PEM regimen, 40 of which underwent comprehensive 72-week body composition analysis. The pretreatment clinical characteristics of the cohort are summarized in **Table 1**. Median age was 57 years, with 35 patients (66.0%) being male. One person was using 0.1 mg/day, another person was using 0.4 mg/day, and the remaining 51 people were using 0.2 mg/day of PEM. Body composition analysis revealed the following median values: body weight 71.0 kg, body mass index (BMI) 26.8 kg/m^2^, fat mass 38.8%, soft lean mass 57.7%, SKM mass 33.0%, and SMI 7.4 kg/m^2^. The elevated complication rates of type 2 diabetes mellites (DM) (49.1%) and hypertension (HT) (41.5%) were typical for the Japanese NAFLD population (19). The median values for AST, ALT, alkaline phosphatase (ALP), gamma-glutamyltransferase (GGTP), and FIB-4 index were 42 U/L, 55 U/L, 226 U/L, 56 U/L, and 1.45, respectively.

**Table 1:** Clinical characteristics of HTG-NAFLD patients treated with PEM.

	Baseline
Age (years)	57 (46-67)
Male	35 (66.0%)
Body composition	
Body weight (kg)	71.0 (58.3-79.8)
BMI (kg/m^2^)	26.8 (25.1-30.0)
Fat mass (%)	38.8 (30.1-42.5)
Soft lean mass (%)	57.7 (54.3-66.4)
SKM mass (%)	33.0 (30.8-39.0)
SMI (kg/m^2^)	7.4 (6.6-8.3)
Complications	
LC	4 (7.5%)
Type 2 DM	26 (49.1%)
HT	22 (41.5%)
Obesity (BMI ≥ 25)	41 (77.4%)
Laboratory data	
Alb (g/dL)	4.5 (4.3-4.8)
AST (U/L)	42 (28-61)
ALT (U/L)	55 (36-78)
ALP (U/L)	226 (183-294)
GGTP (U/L)	56 (39-95)
Platelets (×10^3^/µL)	251 (171-301)
FIB-4 index	1.45 (0.9-2.9)
TG (mg/dL)	181 (126-264)
TC (mg/dL)	198 (169-233)
LDL-C (mg/dL)	114 (98-144)
HDL-C (mg/dL)	41 (31-55)
FBS (mg/dL)	117 (104-149)
HbA1c (%)	6.1 (5.7-6.6)
Cre (mg/dL)	0.80 (0.65-0.90)
CK (U/L)	107 (64-151)

Alb, albumin; ALP, alkaline phosphatase; ALT, alanine aminotransferase; AST, aspartate aminotransferase; BMI, body mass index; CK, creatine kinase; Cre, creatinine; DM, diabetes mellitus; FBS, fasting blood sugar; FIB-4, Fibrosis-4; GGTP, gamma-glutamyltransferase; HbA1c, hemoglobin A1c; HDL-C, high-density lipoprotein cholesterol; HT, hypertension; HTG-NAFLD, hypertriglyceridemia accompanying non-alcoholic fatty liver disease; LC, liver cirrhosis; LDL-C, low-density lipoprotein cholesterol; PEM, pemafibrate; SKM, skeletal muscle; SMI, skeletal muscle mass index; TC, total cholesterol; TG, triglyceride.

### Absence of significant treatment-induced changes in body composition parameters over baseline at 24 and 72 weeks

**Figure 1:**
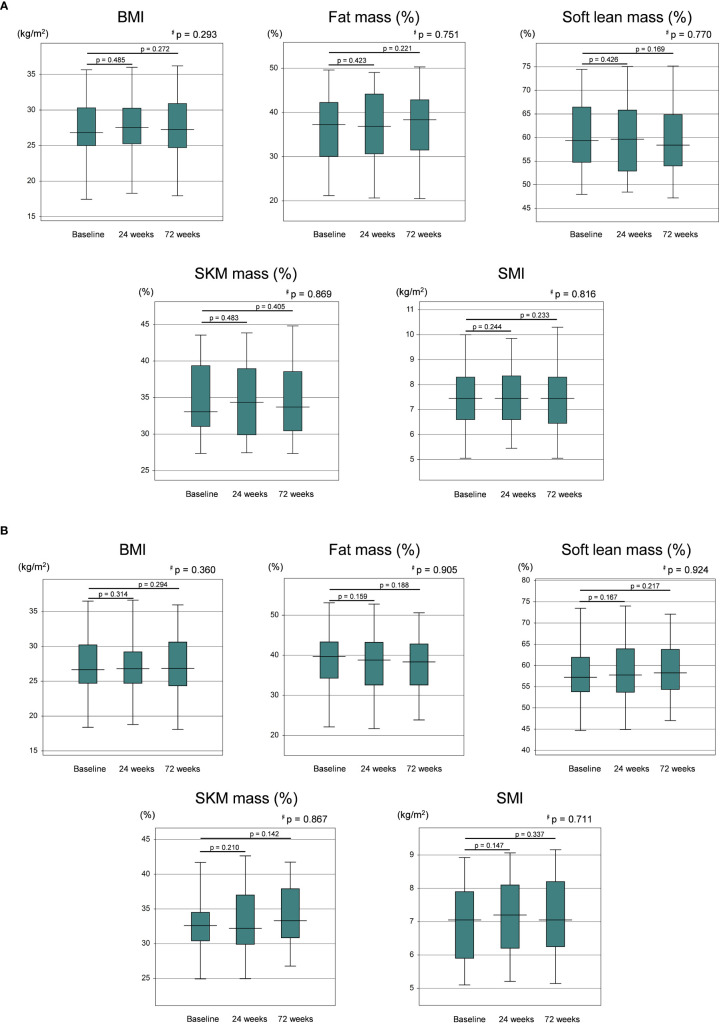
**(A)** Levels of BMI, fat mass (%), soft lean mass (%), SKM mass (%), and SMI at baseline, 24 weeks, and 72 weeks of PEM treatment. **(B)** Levels of BMI, fat mass (%), soft lean mass (%), SKM mass (%), and SMI at baseline, 24 weeks, and 72 weeks of PEM treatment in responders (Wilcoxon matched-pairs signed-rank test). p values are indicated by ^♯^for the Friedman test. BMI, body mass index; PEM, pemafibrate; SKM, skeletal muscle; SMI, skeletal muscle mass index.

PEM can theoretically influence PPARα and its targets throughout the entire body, including adipose tissue and muscle, to alter body composition. However, body composition parameters, including BMI, fat mass (%), soft lean mass (%), SKM mass (%), and SMI showed no significant changes from baseline to 24 weeks and 72 weeks (**Figure 1A**). In a sub-analysis focused on the PEM responders with an improvement of ALT >30%, no statistically significant changes in body composition parameters from baseline at 24 and 72 weeks were observed (**Figure 1B**).

### Seventy-two-week treatment with PEM significantly improved liver function and lipid profiles

We analyzed the changes in clinical parameters from before to 72 weeks of PEM treatment in patients with HTG-NAFLD (**Table 2**). The median values of AST, ALT, ALP, and GGTP all showed significant improvements at 72 weeks over baseline (AST: 42 to 30 U/L, ALT: 55 to 27 U/L, ALP: 226 to 60 U/L, GGTP: 56 to 34 U/L; all p < 0.001). A significant increase in serum albumin (Alb) was observed as well (4.5 to 4.6 g/dL, p = 0.018). Lipid profiles, including TG and total cholesterol (TC), showed improvements at 72 weeks compared with baseline, as previously reported (TG: 181 to 118 mg/dL; p < 0.001, TC: 198 to 187 mg/dL; p = 0.006). No significant changes in Cre values were seen. All patients were able to complete PEM treatment without noticeable adverse effects.

**Table 2:** Characteristics of 53 patients with HTG-NAFLD at baseline and after 72 weeks of PEM treatment.

PEM treatment			
	Baseline	72 weeks	p-value
Alb (g/dL)	4.5 (4.3-4.8)	4.6 (4.4-4.9)	**0.018**
AST (U/L)	42 (28-61)	30 (22-36)	**< 0.001**
ALT (U/L)	55 (36-78)	27 (20-42)	**< 0.001**
ALP (U/L)	226 (183-294)	60 (44-86)	**< 0.001**
GGTP (U/L)	56 (39-95)	34 (25-53)	**< 0.001**
Platelets (×10^3^/μL)	251 (171-301)	248 (194-303)	**0.009**
FIB-4 index	1.45 (0.9-2.9)	1.30 (0.8-2.2)	0.069
TG (mg/dL)	181 (126-264)	118 (88-169)	**< 0.001**
TC (mg/dL)	198 (169-233)	187 (174-206)	**0.006**
LDL-C (mg/dL)	114 (98-144)	117 (93-126)	**0.004**
HDL-C (mg/dL)	41 (31-55)	48 (36-56)	0.097
Cre (mg/dL)	0.80 (0.65-0.90)	0.76 (0.62-0.83)	0.426

Alb, albumin; ALP, alkaline phosphatase; ALT, alanine aminotransferase; AST, aspartate aminotransferase; Cre, creatinine; FIB-4, Fibrosis-4; GGTP, gamma-glutamyltransferase; HDL-C, high-density lipoprotein cholesterol; HTG-NAFLD, hypertriglyceridemia accompanying non-alcoholic fatty liver disease; LDL-C, low-density lipoprotein cholesterol; PEM, pemafibrate; TC, total cholesterol; TG, triglyceride. p<0.05 is indicated in bold.

### Comparison between responders and non-responders to PEM treatment in HTG-NAFLD patients

**Figure 2:**
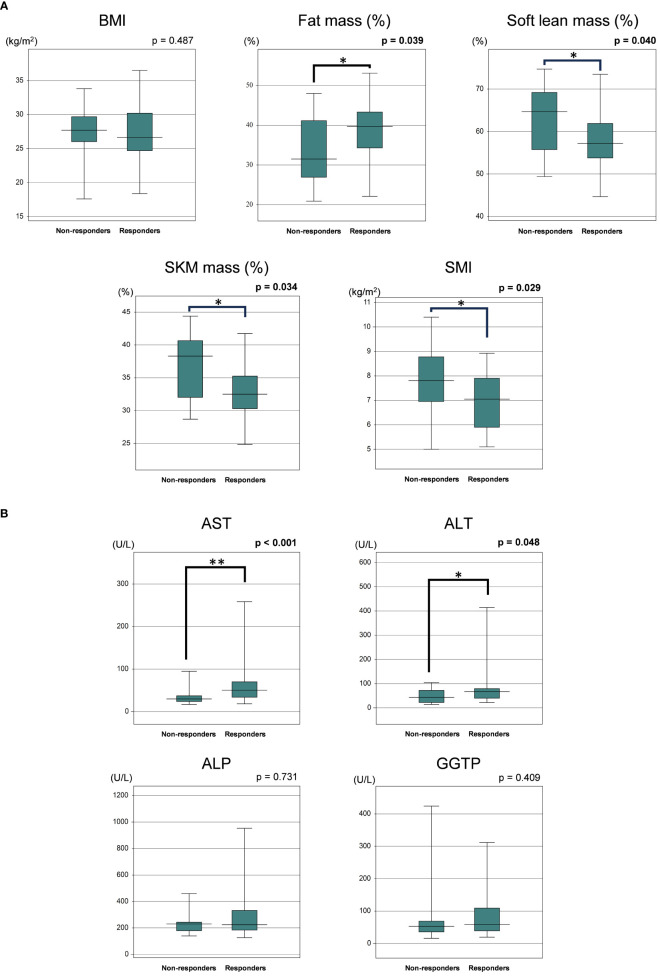
**(A)** Comparison of baseline body composition parameters of responders and non-responders to 72 weeks of PEM treatment. **(B)** Comparison of baseline laboratory data of responders and non-responders to 72 weeks of PEM treatment (Mann–Whitney U test). *p < 0.05, **p < 0.001. ALP, alkaline phosphatase; ALT, alanine aminotransferase; AST, aspartate aminotransferase; BMI, body mass index; GGTP, gamma-glutamyltransferase; PEM, pemafibrate; SKM, skeletal muscle; SMI, skeletal muscle mass index.

We next compared the baseline clinical features of PEM responders with an improvement of ALT >30% (n = 34) and non-responders (n = 19) among the HTG-NAFLD patients. Responders had a significantly higher frequency of female (73.5 vs. 42.1%, p = 0.023). The presence of LC, HT, and type 2 DM did not significantly affect PEM treatment response (**Table 3**). In comparisons of the clinical parameters of responders and non-responders before PEM treatment, body composition analysis revealed that responders had significantly higher fat mass (%), lower soft lean mass (%), lower SKM mass (%), and lower SMI prior to PEM treatment (fat mass: 39.7 vs. 31.5%; p = 0.039, soft lean mass: 57.3 vs. 64.7%; p = 0.040, SKM mass: 32.5 vs. 38.3%; p = 0.034, SMI: 7.0 vs. 7.8 kg/m^2^; p = 0.029) (**Figure 2A**). Responders also exhibited significantly higher levels of AST and ALT before PEM treatment (AST: 49 vs. 30 U/L; p <0.001, ALT: 65 vs. 43 U/L; p = 0.048) (**Figure 2B**).

**Table 3:** Patient background comparison of responders and non-responders to 72 weeks of PEM treatment.

	Non-Responder (n=19)	Responder (n=34)	
	n (%)	n (%)	p-value
Female	8 (42.1)	25 (73.5)	**0.023**
LC	3 (15.8)	1 (2.9)	0.089
HT	8 (42.1)	14 (41.2)	0.947
Type 2 DM	10 (52.6)	15 (47.1)	0.697

Patients were defined as responders if ALT decreased by > 30% at 72 weeks of PEM administration. DM, diabetes mellitus; HT, hypertension; LC, liver cirrhosis; PEM, pemafibrate. p<0.05 is indicated in bold.

**Figure 3:**
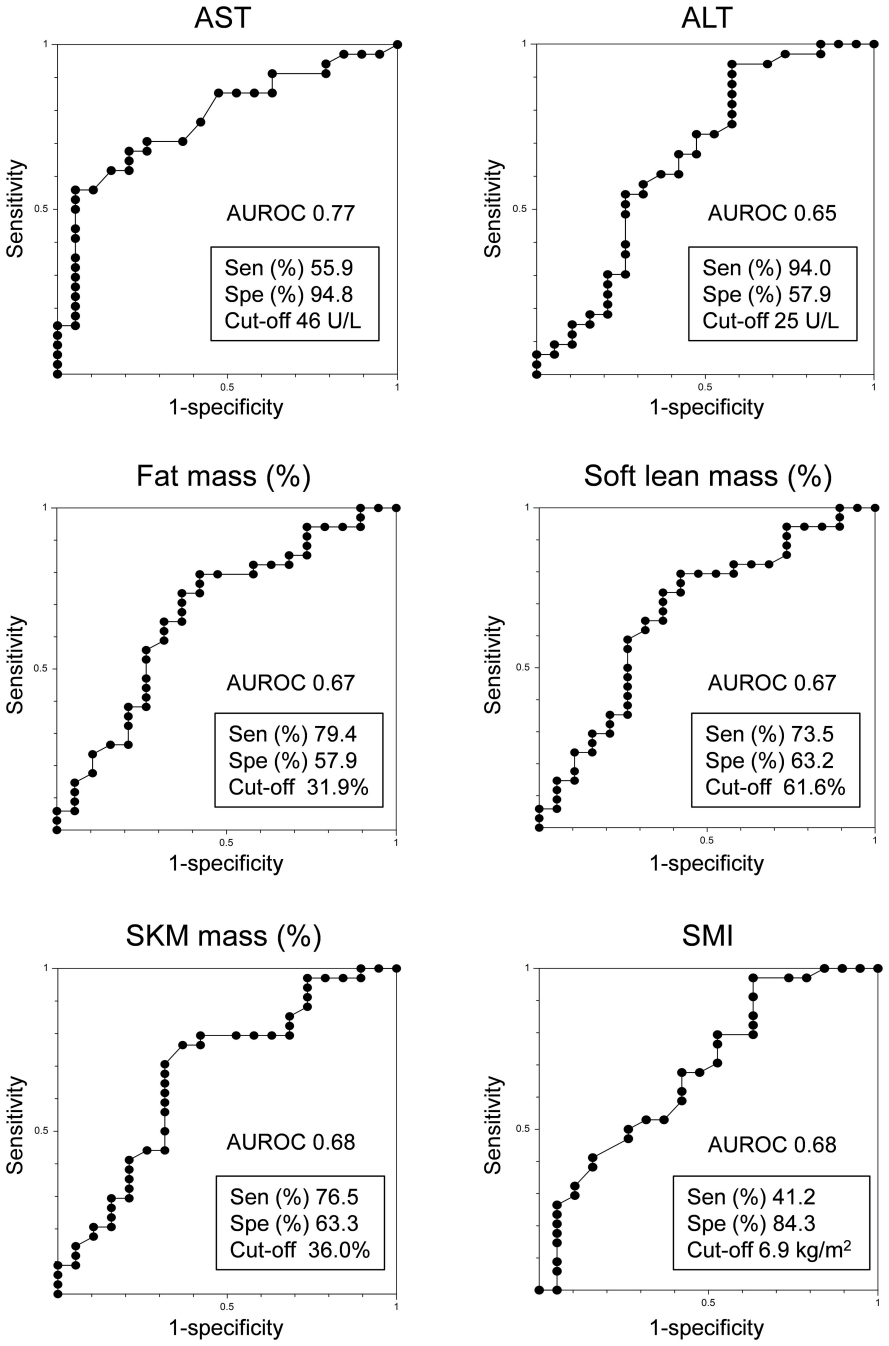
Receiver operating characteristic analysis of PEM responders in HTG-NAFLD patients. ALT, alanine aminotransferase; AST, aspartate aminotransferase; AUROC, area under the receiver operating characteristic curve; PEM, pemafibrate; Sen, sensitivity; SKM, skeletal muscle; SMI, skeletal muscle mass index; Spe, specificity.

The respective AUROC values for AST, ALT, fat mass (%), soft lean mass (%), SKM mass (%), and SMI were 0.77, 0.65, 0.67, 0.67, 0.68, and 0.68, respectively. The most appropriate ROC cut-off values for discriminating between responders and non-responders were AST: 46 U/L (sensitivity: 55.9%, specificity: 94.8%), ALT: 25 U/L (sensitivity: 94.0%, specificity: 57.9%), fat mass (%): 31.9% (sensitivity: 79.4%, specificity: 57.9%), soft lean mass (%): 61.6% (sensitivity: 73.5%, specificity: 63.2%), SKM mass (%): 36% (sensitivity: 76.5%, specificity: 63.3%) and SMI: 6.9 kg/m^2^ (sensitivity: 41.2%, specificity: 84.3%) (**Figure 3**).

## Discussion

This prospectively registered, retrospectively observed cohort assessed the impact of an extended 72-week PEM regimen on body composition in patients with HTG-NAFLD. Similarly to short-term PEM administration (10), no notable alterations in body composition were observed, and consistent outcomes emerged across treatment-responsive subgroups. Regarding the effects of PEM treatment, the intended metabolic shifts were achieved in terms of reductions in AST, ALT, GGTP, ALP, TG, and TC levels along with an increase in Alb levels. Furthermore, this investigation identified that 72-week PEM treatment responders with a > 30% decrease in ALT were predominantly women with higher AST, ALT, and fat mass (%) along with lower soft lean mass (%), SKM mass (%), and SMI, which corroborated previous findings of short-term PEM treatment (10).

The metabolism of liver fatty acids and TG is closely regulated through a delicate balance of *de novo* lipogenesis, glyceroneogenesis, very low-density lipoprotein assembly and secretion, lipolysis, and fatty acid oxidation at both the transcriptional and post-transcriptional levels (20, 21). Multiple studies have suggested that compromised PPARα function and impaired fatty acid oxidation play significant roles in the development of NASH (22, 23). In this context, it is plausible that PEM, which activates PPARα, improves the pathogenesis of NASH. In patients with NAFLD, PEM treatment has been reported to improve liver fibrosis markers along with findings on magnetic resonance elastography, transient elastography, and hepatic shear wave velocity, indicating a positive effect on liver fibrosis (7, 12, 24, 25). Notably, those studies indicated that PEM did not lead to a reduction in BMI and that the therapeutic benefits of PEM were independent of weight loss. Our previous data from a 24-week PEM treatment study revealed that the treatment response to PEM was more favorable in patients with higher body fat (10). We therefore hypothesized that longer term PEM administration would alter body composition by stimulating PPARα and its target genes in adipose and muscle tissue in this 72-week PEM study. In contrast to the discernible alterations observed in liver enzymes and lipid profiles, however, no significant changes were detected in fat mass or other relevant body composition parameters. Our results are consistent with the previous mouse study showing that a clinical dose of PEM treatment does not induce PPARα target genes in extrahepatic tissues, including BAT and eWAT (9).

In earlier reports, the therapeutic effect of PEM was verified by changes in FAST score (26), normalization of ALT (27), a 30% reduction in ALT, and a 30% reduction in shear wave velocity (12). However, investigations of histopathological changes in human liver, adipose, and muscle tissue as well as variations in gene expression with PEM treatment in NAFLD have not been adequately investigated. Exploring the detailed effects of PEM on the human liver both intra- and extrahepatically is a future challenge.

This study had several limitations, primarily stemming from its retrospective, single-center, non-interventional design. To validate our findings, it will be necessary to conduct further large-scale, prospective investigations. And, the gold standard for body composition measurement includes dual-energy X-ray absorptiometry (DXA) and magnetic resonance imaging (MRI). Therefore, it is essential to determine whether the results obtained from the multi-frequency impedance body composition analyzer used in this study are consistent with those obtained from MRI or DXA.

In conclusion, 72 weeks of PEM treatment resulted in improvements in liver enzymes and lipid profile, but not significant changes in body fat mass. Female, higher AST and body fat percentage, and lower soft lean mass percentage were associated with better response to long-term PEM treatment.

## Data availability statement

The raw data supporting the conclusions of this article will be made available by the authors, without undue reservation.

## Ethics statement

The studies involving humans were approved by Committees for Medical Ethics of Nagano Municipal Hospital. The studies were conducted in accordance with the local legislation and institutional requirements. The participants provided their written informed consent to participate in this study. Written informed consent was obtained from the individual(s) for the publication of any potentially identifiable images or data included in this article.

## Author contributions

TI: Writing – original draft, Methodology, Formal analysis, Data curation, Conceptualization. TK: Writing – original draft, Methodology, Investigation, Funding acquisition, Formal analysis, Conceptualization. HKu: Writing – review & editing, Resources, Project administration, Investigation, Data curation, Conceptualization. TO: Writing – review & editing, Investigation, Data curation. S-IW: Writing – review & editing, Software, Resources, Formal analysis, Data curation. HKo: Writing – review & editing, Investigation, Data curation. YY: Writing – review & editing, Methodology, Investigation. AS: Writing – review & editing, Investigation, Data curation. NT: Writing – review & editing, Supervision. TU: Writing – review & editing, Supervision, Resources.

## Glossary

**Table:**

Alb	albumin
ALP	alkaline phosphatase
ALT	alanine aminotransferase
AST	aspartate aminotransferase
Atgl	adipose triglyceride lipase
AUROC	area under the receiver operating characteristic curve
BAT	brown adipose tissue
BMI	body mass index
Cre	creatinine
DM	diabetes mellitus
DXA	dual-energy X-ray absorptiometry
eWAT	epididymal white adipose tissue
FAST	FibroScan-aspartate aminotransferase
FIB-4	Fibrosis-4
FGF21	fibroblast growth factor 21
GGTP	gamma-glutamyltransferase
HCC	hepatocellular carcinoma
Hsl	hormone-sensitive lipase
HT	hypertension
HTG	hypertriglyceridemia
LC	liver cirrhosis
MRI	magnetic resonance imaging
NAFLD	non-alcoholic fatty liver disease
NASH	non-alcoholic steatohepatitis
PEM	pemafibrate
PPARα	peroxisome proliferator-activated receptor-alpha
ROC	receiver operating characteristic
SKM	skeletal muscle
SMI	skeletal muscle mass index
TC	total cholesterol
TG	triglyceride
